# My cells, my model: immune-competent autologous organ-on-chip systems as a new paradigm in precision medicine

**DOI:** 10.3389/fimmu.2025.1712796

**Published:** 2025-12-12

**Authors:** Francesco Bisconti, Hugo Abreu, Thuy Duong Nguyen, Fabiola Stolfi, Noemi Corbezzolo, Giuseppe Gigli, Davide Raineri, Giuseppe Cappellano, Alessandro Polini, Francesca Gervaso, Annalisa Chiocchetti, the FLAMIN-GO Consortium

**Affiliations:** 1 Institute of Nanotechnology, National Research Council (CNR-NANOTEC), Lecce, Italy; 2 Tecnomed Puglia – Technopole for Precision Medicine (Biotech Lecce Hub), Lecce, Italy; 3 Department of Health Sciences, Interdisciplinary Research Center of Autoimmune Diseases-IRCAD, University of Eastern Piedmont, Novara, Italy; 4 Center for Translational Research on Autoimmune and Allergic Diseases, University of Eastern Piedmont, Novara, Italy; 5 Department of Engineering for Innovation, University of Salento, Lecce, Italy; 6 Department of Experimental Medicine, University of Salento, Lecce, Italy

**Keywords:** autologous organ-on-chip, precision medicine, disease modelling, tissue engineering, drug testing

## Abstract

Organ-on-chip (OoC) technology aims to replicate key physiological functions of one or more tissues within sophisticated three-dimensional microfluidic platforms. Beyond their engineering advances, OoC systems are increasingly recognized for their potential to bring preclinical research closer to clinical reality, especially when incorporating patient-derived cells. This autologous dimension represents a new frontier, as it enables the faithful modeling of individual immune processes in a physiologically relevant and truly personalized context. Importantly, if the immune system itself is to be incorporated on-chip, the requirement for autologous integration extends to all tissues involved, ensuring consistency and fidelity of patient-specific responses. Academic and industrial efforts have progressively advanced from single-tissue to multi-tissue and multi-organ OoC systems, converging toward autologous OoC (aOoC) platforms that can (i) capture patient-specific immunopathophysiology with higher fidelity, (ii) potentially complement and, in specific contexts, reduce reliance on animal models, and (iii) directly inform immunotherapy development and therapeutic decision-making within precision medicine. In this review, we first summarize the principles and fabrication strategies underlying OoC technology, then trace their evolution toward autologous systems capable of modeling autoimmune diseases and assessing drug efficacy and safety in a translationally relevant manner. Finally, we discuss the current limitations of these platforms and outline the major challenges that must be addressed to advance their translational potential.

## 1 Introduction

Pathophysiology research and drug development both heavily rely on robust *in vitro* models. Two-dimensional (2D) cell culture has been until now the most common cell culturing technique due to low cost, simplicity, and great compatibility with high throughput analysis. However, the poor fidelity to the *in vivo* conditions limits its efficacy, widening the translational gap between preclinical studies and clinical trials (1–8). The reason behind that is very simple: in 2D cell culture systems, cells are generally grown on rigid plastic surfaces as mono-layer, lacking proper biochemical and physical stimuli (9). On the contrary, in our body, cells are immersed in a three-dimensional (3D) environment and experience biochemical and physical spurs of mechanical and electrical nature (10–15).

Hydrogel-based cell culture systems, spheroids and organoids, or their combination currently represent valid 3D cell culture models (16, 17). These platforms employ highly hydrated 3D polymeric matrices that mimic the native extracellular matrix (ECM) and recapitulate key structural and functional features of tissues, making them useful tools for diagnostic, disease modelling, drug discovery and precision medicine (18–25). Different hydrogels have been reported in the literature, of natural or synthetic origin, usually with the common features to be responsive to physical (*e.g.*, heat, light) or chemical (*e.g.*, pH variation) cues that induce a sol-gel transition (26), and the possibility to tune the physic-chemical characteristics of the material according to the *in vivo* ECM that must be recapitulated. Evolving in complexity, there are organotypic 3D *in vitro* models such as spheroids (24, 27). These are multicellular aggregates formed exploiting different driving forces (*e.g.*, gravitational, centrifugal, etc.) in the presence or the absence of 3D matrices (28, 29). The opportunity to obtain heterogenous architectures, internal gradients of signaling molecules, nutrients, and both chemical and physical stimuli is a key point that makes spheroids a useful tool to study various pathophysiological conditions, with a prominent role in cancer research, from *in vitro* preclinical models to clinical applications (30–32). Lastly, organoids are cell culture systems based on embryonal, adult and human induced pluripotent stem cells. They rely on manipulation of factors involved in the embryonic organogenesis for producing a cell self-organization that leads to tissue- or organ-like structures (33, 34), widely employed in studies of disease modelling, drug screening, cell therapy and personalized medicine (35, 36).

In the context of *in vitro* models, a significant milestone is marked by the development of organ-on-chip (OoC) technology since they offer superior control over the cellular microenvironment, enable dynamic perfusion and mechanical stimulation, support real-time monitoring, and allow multi-tissue integration with higher reproducibility compared to organoids and spheroids (37–40).

**Figure 1:**
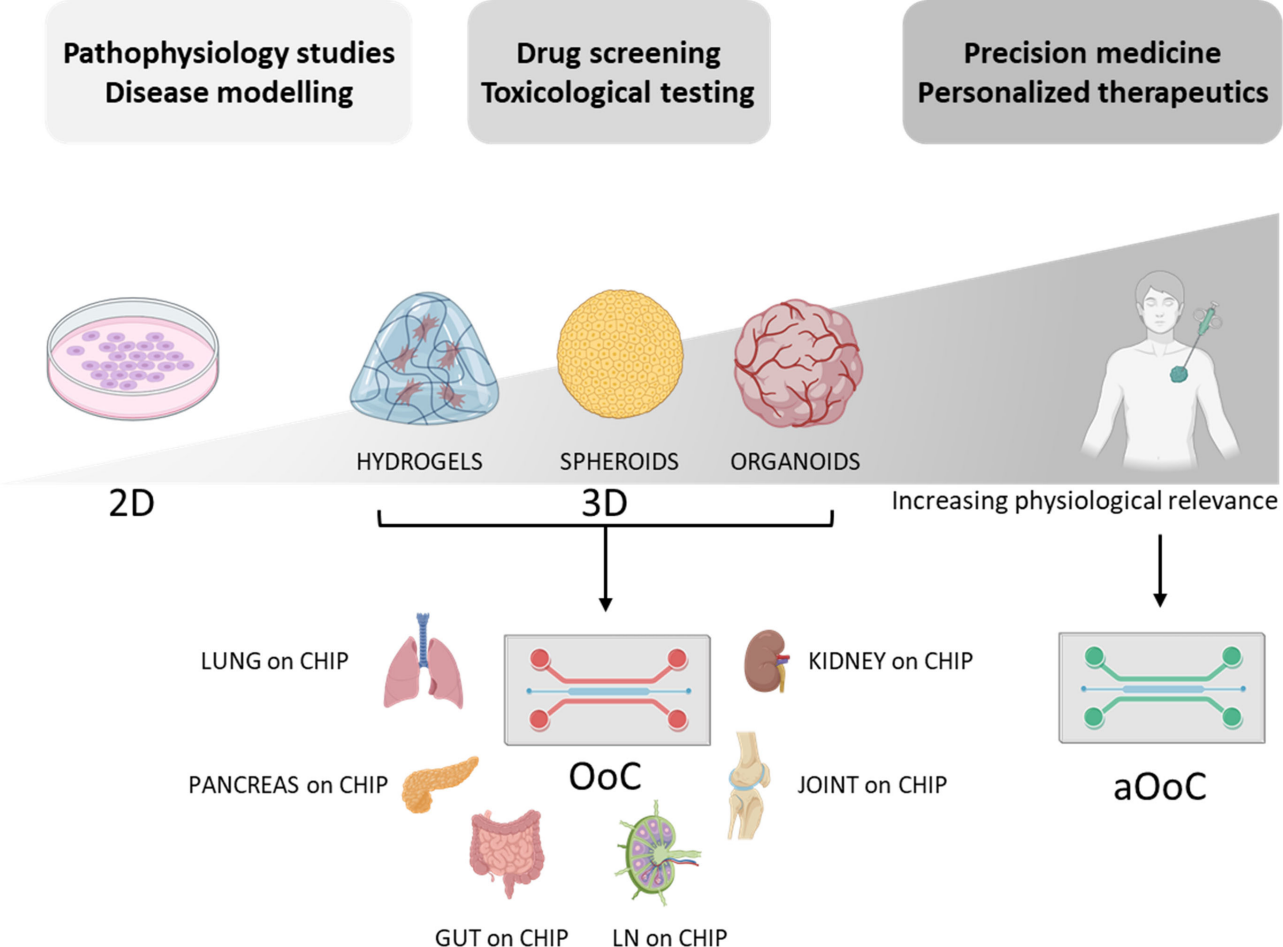
*In vitro* models evolution towards organ-on-chip. Overview of the transition from 2D cell cultures to Organ-on-Chip technologies, incorporating intermediate 3D models such as spheroids and organoids supported by polymeric hydrogel matrices. Conventional OoCs use immortalized cell lines or donor cells to recreate tissue and organ models, while aOoCs employ autologous cells to better predict individual responses to therapeutic agents. This shift supports the advancement of personalized medicine. OoC platforms can be applied in disease modelling, drug screening, toxicological testing, and development of precision therapeutics. Created with Biorender.com.

OoC are microfluidic devices generally fabricated exploiting techniques common to microelectronics, such as photolithography (40). These devices are constituted by one or more chambers in which different cell populations are cultivated and exposed to diverse biochemical and/or physical stimuli, faithfully mimicking what they experience *in vivo* (41–44). Thus, the synergy between OoC and other 3D culture models, such as hydrogels, spheroids, and organoids, is of paramount importance. These diverse technologies can be seamlessly interconnected within OoC platforms, offering sophisticated and physiologically relevant environments, as depicted in **Figure 1**.

By carefully adjusting factors such as substrate stiffness, topographical cues, and fluid flow patterns, OoC technology enables the incorporation of one or more cell types within a single or interconnected device effectively mimicking various organ functions in a controlled environment (45). The design and architecture of these devices are tailored to mimic specific tissues and their respective pathophysiology for targeted studies (46, 47).

Therefore, the ability to replicate human physiological complexity more accurately by modulating the 3D microenvironment, and applying various physical stimuli, underscores the significant value of OoC technology across several pivotal areas of research, from pathophysiology studies to personalized therapeutics.

This review aims to provide a comprehensive overview of OoC technology, with a particular focus on the emerging field of autologous systems. It covers the fundamental principles of OoC, explains how these systems are designed and fabricated, and highlights the technological advancements that underpin their development. The review is structured to be accessible to a multidisciplinary audience, biologists, chemists, clinicians, and engineers, who are new to the field and seeking to understand its potential applications, challenges, and opportunities for contributing to its advancement.

## 2 Organ-on-chip in the 3D *in vitro* models framework

OoCs allow researchers to model human diseases in the lab using immortalized or donor-derived cells cultured in organ-specific microenvironments. This enables the recreation of disease conditions and monitoring of their progression at both organ and multi-organ levels. Hence, OoCs are increasingly used to study disease mechanisms, discover biomarkers, and test therapeutic candidates with improved accuracy compared to traditional cell cultures or animal models (48). The incorporation of patient-derived cells has led to the development of autologous OoC (aOoC), which preserves the unique genomic, epigenomic, and phenotypic traits of the donor (**Figure 1**). This advancement allows investigation of patient-specific disease mechanisms, therapeutic responses, and drug sensitivities (49). In this context, aOoC is emerging as a revolution in both *(i)* drug development process and *(ii)* precision medicine approach (49–54).

*Drug Screening and Toxicology Testing.* Drug development continues to face marked inefficiencies, with approximately 90% of drug candidates failing to reach approval despite extensive preclinical testing (55). Traditional 2D *in vitro* models, although cost-effective and suitable for high-throughput studies, lack the architectural complexity and biochemical microenvironment of human tissues. Animal models, while more physiologically complex, are expensive, ethically constrained, and often poorly predictive of human pathophysiology, contributing to translational failure (56). Biomarker-guided clinical trials represent a promising evolution but remain costly and require long timelines to identify meaningful patient responses (55).

OoC platforms provide a human-relevant alternative by enabling the assessment of drug efficacy, toxicity, and pharmacokinetics under dynamic, physiologically inspired conditions. As reported by Marrella et al., an *in vitro* dynamic fluidics model of ovarian cancer using SKOV-3 human cell line achieved a similar tumor regression curve and drug efficacy outcomes five times faster compared to a xenograft mouse model. Such examples demonstrate the potential of OoC systems to become a fast, reliable and ethically appropriate approach for drug testing in detriment of conventional *in vivo* models (57). Furthermore, when integrated into multi-organ circuits, they allow the study of systemic drug distribution and cross-organ toxicity in ways conventional cell culture cannot (56, 58, 59). Several studies have already demonstrated improved predictive power in OoC-based drug screening (60, 61), and the U.S. Food and Drug Administration has formally acknowledged their potential by including OoCs within New Approach Methodologies (NAMs) for regulatory science (48).

aOoC models extend these benefits by embedding patient-derived tissues, including primary cells, spheroids, or organoids, within microfluidic platforms that recapitulate organ-specific physiology. By capturing patient-specific genetic and physiological features absent in immortalized cell lines, aOoCs allow more accurate evaluation of individual drug sensitivity and toxicity, providing a strategic avenue to accelerate and refine therapeutic development.

*Precision Medicine and Personalized Therapeutics.* Beyond improving predictive drug screening, aOoC systems are highly relevant to the implementation of precision medicine strategies (49, 54). Inter individual genetic variability strongly influences treatment response, and pharmacogenomic differences can significantly affect drug efficacy and safety (62). Using patient derived material, including primary cells, organoids, adult stem cells, or induced pluripotent stem cell derived tissues, enables the creation of platforms that closely replicate each patient’s biological and disease profile.

These autologous systems provide the opportunity to assess pharmacokinetic and pharmacodynamic relationships and guide therapeutic decisions by anticipating individual absorption, distribution, metabolism, excretion, and toxicity (ADMET) profiles. Such approaches are particularly advanced in oncology, where patient specific tumor on chip platforms are increasingly applied to identify optimal treatment strategies and uncover mechanisms of drug resistance (50, 53). As a result, aOoC systems represent a powerful tool for personalizing therapy and advancing mechanistic understanding in a patient specific manner.

Conceptually, aOoC whose cells derive from representative patient subgroups or from responder versus non-responder cohorts could be used to identify treatment response biomarkers. Once established, this approach would enable the testing of multiple drugs on recreated microtissues derived from drug naïve patients, allowing patient stratification based on specific biomarker detection and quantification and guiding clinicians in selecting alternative therapeutic options when the initial treatment proves ineffective. Importantly, in conditions where the affected tissue is directly involved in disease pathogenesis and can be easily obtained via minimally invasive biopsy, the power of this strategy increases substantially. This represents a patient-specific clinical trial-on-chip (CToC) capable of providing an accurate individual drug response prediction without relying on a trial and error approach, thereby reducing healthcare costs and improving therapeutic efficacy.

A specific application of this strategy could be in the field of autoimmune diseases, for rheumatoid arthritis (RA). RA is one of the most prevalent autoimmune diseases worldwide, affecting nearly one percent of the global population, and is characterized by complex, systemic immune dysregulation that primarily targets the synovial joints (63, 64). Currently, there is no definitive cure for RA, although remission of symptoms is more likely to occur when treatment begins early with disease modifying antirheumatic drugs (DMARDs), specifically methotrexate (MTX), the most common first line therapy (65, 66). These treatments can slow disease progression and prevent permanent joint destruction. However, treatment failure occurs in around half of RA patients (67). In such cases, second line therapeutics typically consist of biologic DMARDs designed to modulate specific cellular and molecular inflammatory pathways, targeting B cells (anti-CD20 – rituximab), pro-inflammatory cytokines (tumor necrosis factor [TNF]-α – adalimumab) or its receptors (interleukin-6 receptor [IL-6R] – tocilizumab), among others (66). The administration of non-effective therapies can be detrimental, leading to significant adverse effects involving multiple organs including lung, liver, kidney, skin and gastrointestinal system, and allowing the disease to further progress unchecked (68). Given the wide availability of anti-RA drugs that only prove effective after several cycles, prior individualized target screening to guide treatment choice, particularly through aOoC technology, would enable faster and more precise evaluation of therapeutic response and support a more personalized approach to disease management.

Despite the challenges in sourcing patient-specific cells, scaling production, and maintaining culture viability, significant advancements are ongoing in the field to optimize aOoC systems. Efforts include refining culture conditions, integrating organoid-based approaches, and employing microengineering techniques to boost primary cell functionality and longevity. For instance, researchers are developing methods to sustain cell phenotypes and enhance tissue architecture, while leveraging biomaterials and microfluidic dynamics to mimic the native microenvironment (69–71). These innovations are pivotal for extending the practical lifespan of OoC models, ultimately enabling more accurate disease modeling and personalized therapeutic testing.

## 3 Fabrication technology

OoC devices can be developed using various technologies, each with its own set of advantages and limitations in terms of complexity, cost, reliability, flexibility, scalability, and time efficiency. Below, we highlight some of the key fabrication methods employed for producing OoC devices.

### 3.1 Optical lithography

The most common technologies for fabricating microfluidic systems for biomedical applications, including OoC, rely on optical lithographic processes (72). Since their first application for microelectronic and microelectro-mechanical systems (MEMS), optical lithography, often in combination with soft lithography approaches, has been largely employed for producing microfluidic devices, such as Lab-on-Chip (LoC) and OoC (73–76). A detailed description of the photolithographic process is elsewhere reported (77). Briefly, a desired patterned mold (master) is created by the photoirradiation, through a patterned hard mask, of a light sensitive material (photoresist) deposited on a silicon wafer substrate. Once generated the master piece, a replica molding step allows device production, using a thermoset transparent biocompatible polymer such as poly(dimethyl siloxane) (PDMS). PDMS is the most common and used material for OoC, it is generally poured onto the master and cured in oven. Advantages of this technology reside in the robustness of the process and the achievable resolution that, for standard procedures, well matches the cell-scale (below to a few micron). By contrast, the access to clean room facilities is mandatory for producing master molds, requiring high costs in term of trained personnel and resources. Moreover, most PDMS-based OoC rely on irreversible closing systems, impeding the opening of the chip before endpoint analysis, thus limiting sample accessibility and post-culture interventions (78). In addition, PDMS is known to adsorb hydrophobic small molecules, including many drugs (i.e. bepridil, verapamil) and signaling compounds (i.e. hormones like some estrogens), which can significantly alter concentration profiles during drug screening or pharmacokinetic assays (79–82). To mitigate this limitation, several strategies have been proposed, such as surface functionalization/coating (e.g., PEGylation, or sol–gel layers) and dynamic pre-saturation of PDMS with the compound of interest, or the use of alternative fabrication methods and materials, like thermoplastics (e.g., cyclic olefin copolymer, polymethyl methacrylate) or 3D-printed resins, that exhibit reduced molecular adsorption (79, 83–86).

### 3.2 Hot embossing and injection molding

Two alternative ways for slightly lowering the production cost are represented by hot embossing and injection molding. Hot embossing is a microfabrication technique used to replicate microstructures from a mold onto a substrate using heat and pressure. This method is suitable for creating microfluidic channels and patterns on polymer substrates. Injection molding is a versatile and widely used manufacturing process in which a material is injected into a mold cavity under high pressure. Both techniques are compatible with polymeric materials like Poly(methyl methacrylate) (PMMA). Parameters like temperature and pressure must be well optimized but can still allow a high resolution (87, 88). These techniques are particularly well-suited for mass-producing microfluidic devices with complex geometries; however, they are less ideal for prototyping due to their slower speed and higher costs.

### 3.3 3D printing

An innovative approach for producing OoC is based on the 3D printing (89, 90). Among different technologies available, VAT polymerization techniques like Stereolithography and Digital Light Processing (DLP) are good candidates, as reported in literature (91, 92). In the 3D printing, a CAD of the chip geometry is produced, transferred to the printer and the object is produced layer-by-layer by the photopolymerization of UV-responsive resins. The choice of biocompatible and almost transparent resins allows high cell viability and proliferation without disturbing the optical properties of the device. Although commercially available 3D printers offer resolutions below 100 µm, less precise than those achievable with photolithography, this level of resolution is adequate for cell culture studies and, for certain applications, even advantageous due to greater flexibility and accessibility. Indeed, the great advantages of the 3D printing production of OoC are the low production cost and high versatility, leading to an impressive fast prototyping capability for an easy customization (93).

## 4 Device architecture and conceptualization

A well-defined OoC architecture is essential for faithfully mimicking the structural and functional features of native tissues. Factors to be considered are: *i)* the target tissue and pathophysiology to be modeled; *ii)* the production technology to be exploited, which dictates the materials to be used and resolution; *iii)* the presence or absence of a hydrogel in which the cells can be embedded; *iv)* the integration of physical stimuli; *v)* the analysis that would be carried out (94). Therefore, at this stage, the number and dimension of chambers where cells grow, and of the channels that allow the communication among different cell populations and provide the cell culture medium, are defined. Interestingly, cell seeding within the microfluidic platform may be performed either through manual dispensing or by employing advanced bioprinting technology. The use of bioprinting not only improves the precision and reproducibility of cell placement but also enables the creation of more intricate and reliable cellular structures, as recently demonstrated (59, 95–98).

Physical stimuli can also be added to better mimic the human environment. Mechanical stimuli, like shear stress, tensile stress, compressive loading, have been implemented, showing their influence on cell culture (43, 99, 100). In addition, electrical stimulation has also been explored, particularly in models of electrically excitable tissues, such as cardiac and neural systems, where it promotes cell alignment, maturation, and functional activity (101, 102).

**Figure 2:**
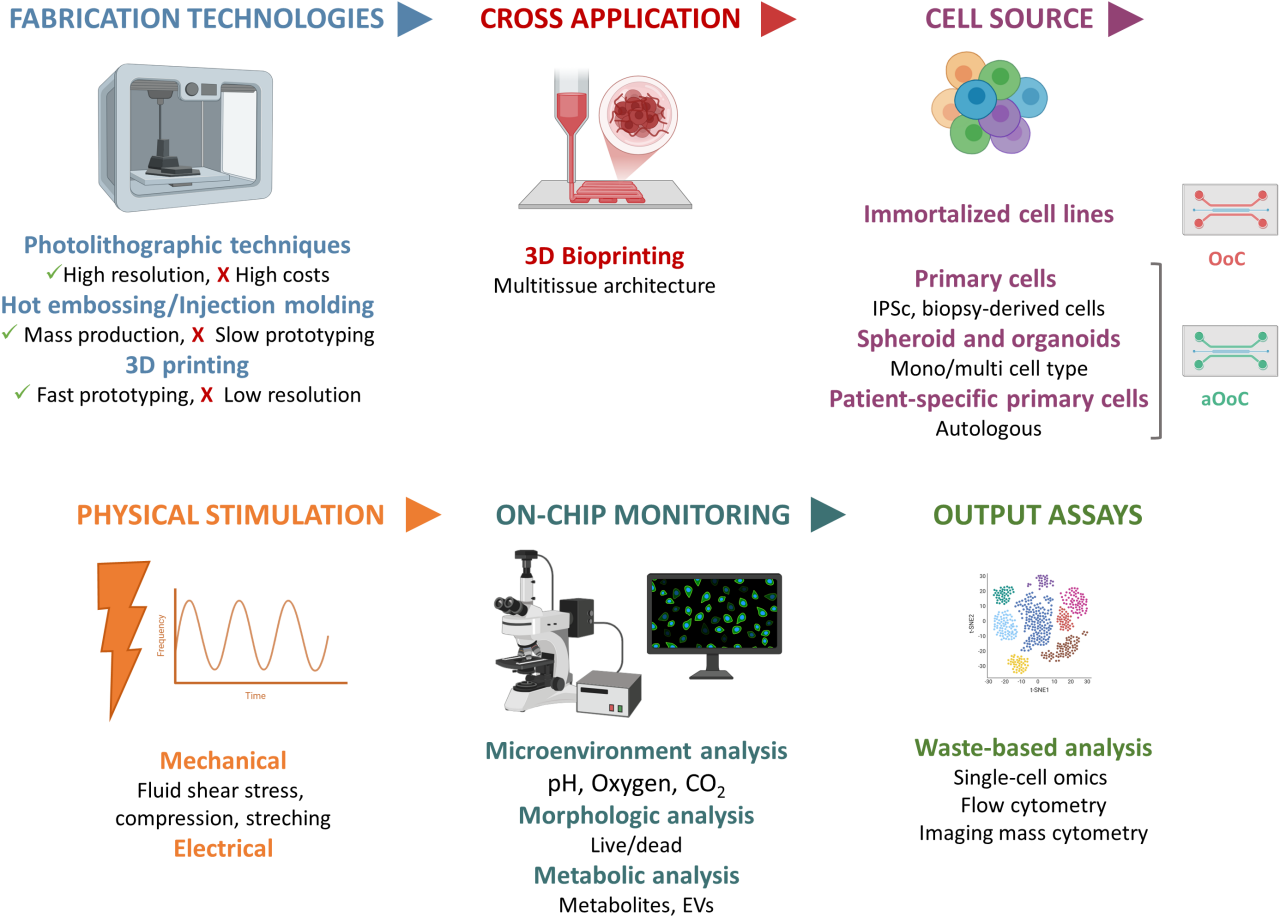
Technological ecosystem surrounding OoC platforms. Schematic representation of the key technologies and methodologies involved in the design, fabrication, stimulation, monitoring, and analytical assessment of OoC systems, spanning from engineering approaches to biological output assays. Created with Biorender.com.

Another important challenge in OoC development is the integration of on-chip monitoring capabilities. Optical and fluorescence-based approaches (i.e. for oxygen, pH, nutrients and metabolites like albumin and glutathione S-transferase) have been widely used (103–109) Then, several other sensing strategies have been explored, such as electrochemical biosensors (i.e. for oxygen, glucose, lactate, temperature) (110–113) impedance- or trans-epithelial/endothelial electrical resistance- (TEER) based measurements (114), and electrophysiological microelectrode arrays (MEAs) for monitoring excitable tissues (115). However, these approaches still present several limitations, including electrode fouling and signal drift during long-term culture, interference from complex cell-culture media, and limited spatial resolution along the xyz axes (104, 116, 117). Despite these challenges, continuous monitoring of oxygen levels, pH, nutrient availability, and cellular metabolites offers valuable insights into the dynamic behavior of the OoC model. Such real-time analyses enable the detection of physiological changes, support the evaluation of cellular responses to drugs or external stimuli, and allow fine-tuning of the microenvironmental conditions to reproduce *in vivo* tissue functionality more accurately. Therefore, as sensor integration in OoC platforms increasingly enables continuous and multimodal data acquisition, a further emerging challenge concerns data interoperability and management. OoCs often combine heterogeneous information streams, such as sensor readouts (i.e., oxygen, pH, metabolites), microfluidic parameters (flow rate, shear stress), and cellular or imaging data, which are typically stored in disparate formats, hindering reproducibility and comparison between studies. To overcome this issue, it is mandatory moving towards standardized data frameworks and open data ontologies that enable the integration and reuse of experimental data (118). These frameworks could also support the integration of experimental outputs with computational models and digital twin platforms, enabling advanced data analytics, cross-laboratory reproducibility, and multi-scale modeling of physiological processes. The integration of machine learning and advanced imaging analysis techniques with OoC platforms has just been explored to further enhance their capabilities (119, 120). **Figure 2** summarizes the input and output technologies utilized in OoC platforms, illustrating the multifaceted approach for the fabrication and integration of monitoring and analysis tools within these advanced *in vitro* models.

## 5 Organ-on-chip: a new paradigm in disease modelling

**Figure 3:**
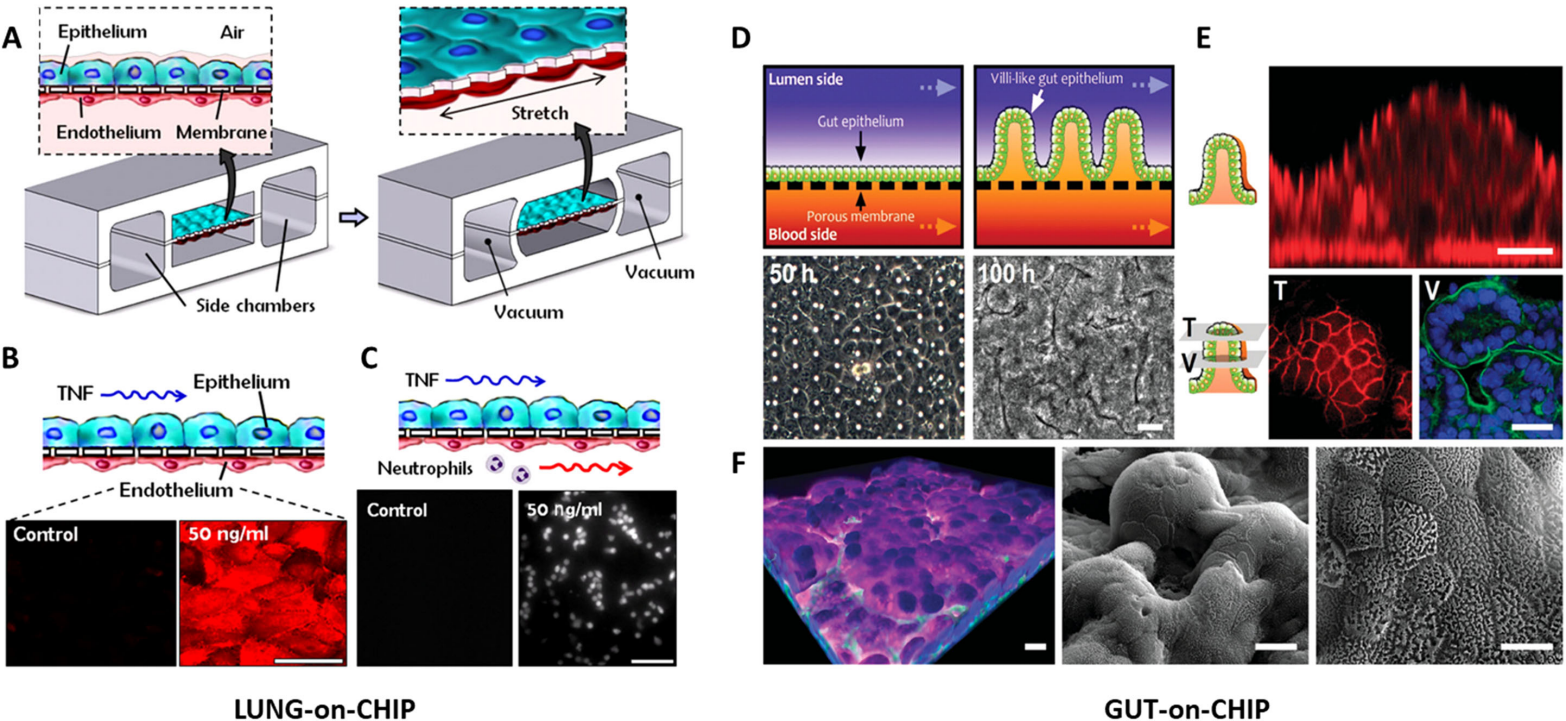
Organ-on-chip models*: The first lung-on-chip*: **(A)** device architecture characterized by two chambers separated by a stretchable porous membrane where alveolar epithelial and ECs are seeded; **(B)** Cell stretched with 10% strain at 0.2 Hz and treated with TNF-α (50 ng/ml), which induces ICAM-1 expression (red) on the endothelium; **(C)** fluorescently labeled human neutrophils (white dots) adhere on the activated vascular cells. Reproduced with permission (122). *Intestine-on-chip*: Formation of intestinal villi by Caco-2 cells within the Gut-on-a-Chip cultures. **(D)** A schematic illustrates the transformation of a flat intestinal epithelium into a villus structure (top), alongside phase contrast images of Caco-2 cells undergoing similar villus morphogenesis, captured at 50 and 100 hours (bottom); **(E)** The left panel presents schematics of a vertical cross section and horizontal cross sections taken from the tip (T) and middle (V) regions of a single villus. On the right, immunofluorescence confocal images of Caco-2 villi highlight staining for the tight junction protein ZO-1 (red) in both vertical (top) and horizontal (bottom left) sections. The bottom right image shows the apical brush border membrane, marked by continuous F-actin staining (green), overlying well-aligned intestinal cell nuclei (blue); **(F)** On the left, a 3D reconstruction of Z-stacked images shows Caco-2 villi stained for nuclei (blue), F-actin (green), and mucin 2 (magenta). Scanning electron microscopy (SEM) images of the villi are displayed in the middle and on the right, at low and high magnifications, respectively. Reproduced with permission (123).

As previously established, OoC technology excels at accurately replicating tissue organization and interactions, as demonstrated by various examples available in literature (121). The pioneering OoC device, based on immortalized cell line, introduced by Ingber’s group to the scientific community in 2010, successfully replicated the alveolar-capillary interface, underscoring the foundational importance of this technology (122). Such innovative microfluidic platform consisted of two parallel microchannels separated by a porous membrane lined, on the opposite sides, with human lung epithelial cells and endothelial cells (ECs). Then, the incorporation of two larger lateral microchambers allowed the stretching of the membrane, mimicking the natural pressure driven stretching that occurs during inspiration (**Figure 3A**). The reliability of the device in replicating the whole-organ responses was evaluated simulating a pulmonary inflammation. Stimulation of the epithelium with TNF-α led to a marked upregulation of Intercellular Adhesion Molecule 1 (ICAM-1) expression in the ECs within 5 hours, subsequently enhancing the adhesion of fluorescently labeled human neutrophils perfused through the vascular microchannel, which is an inflammatory response characteristic of lung tissue during acute inflammation (**Figures 3B, C**). After this first work, many other devices have been developed to mimic lungs by different research groups finding application in disease modeling, toxicology studies and drug testing (89, 124–129). Lung-on-chip also revealed to be an ideal platform for infectious diseases study, where the complex and dynamic device microenvironment generates essential data not achievable with classic 2D *in vitro* models (130, 131). Besides, Dasgupta and colleagues exploited a lung-on-chip to recapitulate the acute exposure to high-dose gamma radiation, as a consequence of radiological disasters or cancer radiotherapy, and cause of radiation-induced lung injury (RILI) (127). An alveolar-capillary interface was successfully reproduced and exposed to gamma radiation. A deep investigation was carried out to evaluate the reliability of the model, analyzing cell morphology, peripheral blood mononuclear cells (PBMC) recruitment, cytokine production, as well as the transcriptomic analysis, highlighting the relevance of the chip. The hypertrophy of the alveolar cells, the increased permeability and the gene expression variation were recorded with the developed lung-on-chip, while the transwell-mediated coculture failed to recapitulate what occurs *in vivo*. Thus, considering the limitations encountered with animal models, like a low grade in mimicking the dose relevant sensitivities (132–134), the proposed OoC system offered many advantages compared to classic *in vitro* or rodent models. In settings where non-human primates are unfortunately still the primary model (135), the microfluidic platform provided a relevant alternative for studying the molecular basis of RILI and the relative therapeutic approaches.

Other examples of successful OoC are found in literature: for example, intestine-on-chip models represent a powerful tool for studying intestinal biology, disease mechanisms, drug responses, and for developing personalized medicine applications. The human intestine is involved in digestion, nutrient absorption, and immune functions, making it pivotal for understanding various physiological processes and diseases (136, 137). Most of the intestine-on-chip models developed so far have been based on immortalized cell lines (123, 138–140). Kim et al. designed an OoC device consisting of microfluidic channels with a flexible membrane, coated with ECM, where human intestinal cells (Caco-2) were seeded (**Figures 3D-F**) (123). The *in vivo* microenvironment was recreated by the fluid flow rate, able to produce low shear stress over the microchannels, and by exerting cyclic strain that mimics physiological peristaltic motions. In this way, a polarized columnar epithelium was obtained into folds that recapitulated the structure of intestinal villi, forming a high integrity barrier to small molecules that better mimics whole intestine than cells cultured in static transwell-based models. Moreover, *Lactobacillus rhamnosus* GG, a common intestinal bacterium, was successfully co-cultured on the luminal surface of the intestinal epithelium, without compromising epithelial cell viability. However, incorporating a complex microbiota-on-chip presents challenges like simulating the anaerobic-to-aerobic gradient, maintaining high microbial diversity and using oxygen gradients to culture both anaerobes and aerobes simultaneously. Approaches to overcome this challenge include design of microfluidic devices with precisely controlled oxygen gradients, incorporating nanoporous membranes separating microbial and host cells while permitting molecular exchange. Marzorati et al. designed a Host Microbiota Interaction (HMI) module, that permits analysis of aerobic and anaerobic microbes, including complex living microbiome derived from a human intestinal microbial ecosystem (SHIME) reactor, where both microbes and human Caco-2 cells were co-cultured under an oxygen gradient (141). In this device, microbes were separated from the human cells by a nanoporous membrane with an artificial mucus layer, and even under these conditions, the co-cultures were only maintained for 48 hours. Firoozinezhad et al. integrated microscale oxygen sensors into their intestine-on-chip to enable real-time *in situ* measurement of oxygen levels (142). By placing the chips within a custom-engineered anaerobic chamber, they established a physiologically relevant oxygen gradient across co-cultured human intestinal epithelial and microvascular ECs. Both cell types were grown in parallel microchannels separated by a porous, ECM–coated membrane, under controlled oxygen conditions. This device allowed simultaneous monitoring of oxygen levels and intestinal barrier integrity, maintaining physiologically relevant conditions for at least five days *in vitro*. As an alternative to introducing live bacteria into OoC systems, which can present technical and biosafety challenges, microbial-derived extracellular vesicles (EVs) could be used to study host responses. Moreover, EVs derived from probiotic strains may represent a promising approach to investigate beneficial microbe–host interactions within chip-based models (143).

## 6 Autologous organ-on-chip: one step closer to personalized medicine

OoC models utilize microfluidic platforms to replicate organ functions within a controlled environment. aOoC models advance this approach by incorporating patient-specific cells, deriving from biopsy tissues, thereby creating personalized systems that more precisely reflect an individual’s unique physiology and pathology. Cells derived from patient biopsies retain key epigenetic characteristics for several passages in culture, preserving aspects of their original tissue identity and disease-specific signatures. This epigenetic memory can influence gene expression profiles and cellular behavior, making primary cells valuable for modeling patient-specific physiology and pathophysiology *in vitro*. However, prolonged passaging may lead to gradual epigenetic drift, highlighting the importance of using early-passage cells for high-fidelity disease modeling and drug testing (144).

aOoC models offer unique value for precision medicine, since they support the development of therapeutic strategies that are finely adapted to the biology of each individual patient. By incorporating cells that retain the molecular and functional features of the native tissue, these platforms allow researchers to reproduce patient specific disease mechanisms and treatment responses *in vitro*. Although the number of published studies is still limited, the integration of patient derived primary cells and organoids into OoC systems is emerging as a powerful approach to advance personalized therapy design and mechanistic disease investigation (145). An overview of the main cellular models currently available for building OoC platforms, including their advantages and limitations, is provided in **Table 1**.

**Table 1:** Comparative overview of cell sources for OoC applications: potential and pitfalls.

Cell source	Typical yield	Time to generate functional cells/Tissues	Phenotype stability	Representative uses in aOoC	Key advantages	Key limitations	References
*Peripheral Blood Mononuclear Cells (PBMCs)*	Moderate Approx. 1x10^6^–3x10^6^ cells per mL blood	Rapid a few days for immune differentiation	Moderate short-term stability; prone to activation or senescence	Autoimmune disease models, tumor-on-chip, immunotoxicity assays	Minimally invasive; readily available; directly reflects immune state	Difficult to maintain long-term phenotypes	(146–148)
*Induced Pluripotent Stem Cells (iPSCs)*	High expandable indefinitely after reprogramming	Long 1–2 months for reprogramming and differentiation	Variable depends on differentiation protocol and culture conditions	Multi-organ chips, virtually every adult cell type can be replicated with iPSCs	Unlimited supply; patient-specific genetic background; multi-lineage potential	Time- and cost-intensive; potential for epigenetic drift or incomplete maturation	(147, 149, 150)
*Primary differentiated cells*	Variable depends on biopsy site and tissue type	Short Days to weeks, might require purification/selection	High *in vivo*-like phenotype	Disease modeling of one specific tissue	Minimal reprogramming required; faithful to the tissue of origin	Limited proliferative capacity; may dedifferentiate in culture over time	(47)
*Mesenchymal Stem Cells (MSCs)*	Moderate/High Depending on the tissue of origin, adipose typically grant higher yield than bone marrow	Moderate 4–6 weeks for selection, expansion, characterization and differentiation	Moderate stable for limited passages	Regenerative and inflammation-related aOoC models; bone/muscle/chondral tissues	Regenerative potential	Limited lineage diversity; batch-to-batch variability	(35, 151, 152)
*Organoids (patient-derived)*	Moderate expandable over several passages	Moderate 2–6 weeks to mature organoids	High retain 3D structure and many *in vivo* characteristics	Complex tissue modeling gut-on-chip, tumor-on-chip, brain-on-chip	3D architecture; multicellular composition; good mimic of native microenvironment	Variable reproducibility; integration with microfluidics can be challenging	(153–155)

aOoC models have been predominantly applied in cancer research, as tumors are relatively accessible for biopsy, providing the isolation of patient-specific cells (156). These platforms allow researchers to replicate tumor microenvironments, support personalized drug testing, and enable the investigation of cancer progression mechanisms. Nonetheless, almost any tissue amenable of biopsy, such as skin, liver, intestine and lung, can serve as a cell source to create patient-specific OoC models. Noteworthy, blood is also easily accessible and can be used to isolate immune cells, enabling the development of inflamed aOoC models to study immune-related disorders. These models facilitate personalized investigations into autoimmune diseases, infections, and individual responses to immunotherapies (157–159).

### 6.1 Autologous lung-on-chip

Evolving from the first developed model, patient-derived lung epithelial primary cells, offer a unique opportunity to recreate the patient’s physiological environment *in vitro*. These cells can be directly isolated from patient pulmonary biopsy samples and cultured on microfluidic devices, mimicking the microarchitecture and function of specific organs like the lung. Moreover, patient-specific lung organoids, derived from induced pluripotent stem cells (iPSCs) or adult tissue stem cells, cultured into microfluidic devices, also allow for the creation of physiologically relevant systems that better represent individual patient characteristics (71, 160). Jung and colleagues introduced a novel microfluidic platform designed to cultivate 3D lung cancer organoids and perform drug sensitivity assays within a single micro-physiological system (161). This device, realized by soft-lithography methods, successfully generated size-controllable organoids supporting the growth from primary small-cell lung cancer (SCLC) tissues. The study demonstrated that organoids treated with cisplatin and etoposide, standard chemotherapy agents for lung cancer, exhibit concentration-dependent apoptosis. Although the presence of chemo-resistant cells in the core of the organoids highlighted the complexity of tumor microenvironments, and the challenge of overcoming drug resistance, this platform revealed to have high predictive value for therapeutic efficacy of anti-cancer drugs.

Differently, van Riet and colleagues developed a methodology where primary alveolar type 2 (AEC2) cells are first cultivated as organoids to enrich and expand the cell population before their subsequent integration into a microfluidic device (160). They started by dissociating emphysematous lung tissue to obtain a cell suspension. They produced and characterized AEC2 organoids by the presence of the alveolar marker HTII-280 and the absence of the airway basal cell marker keratin 5 (HTII-280^+^/KRT5^-^). These organoids were confirmed to contain intact lamellar body-like structures both within the cells and in the lumen of the organoids, validating their alveolar identity. Following this characterization, the organoids were dissociated, and the enriched AEC2 cells were seeded onto an OoC system, enabling their application in a microfluidic environment.

This strategy holds considerable promise for precision medicine, as it supports therapies adapted to each patient’s unique cellular and genetic profile. However, current data remain limited, and additional studies are needed to further develop autologous organ on chip platforms for lung diseases and other systems, including immune related conditions.

### 6.2 Autologous lymph node-on-chip

Immune responses differ markedly from person to person, making it essential to understand patient specific drug effects and disease mechanisms. aOoC platforms offer a promising route to recreate individual immune environments and support the development of tailored therapies and new immunomodulatory drugs.

**Figure 4:**
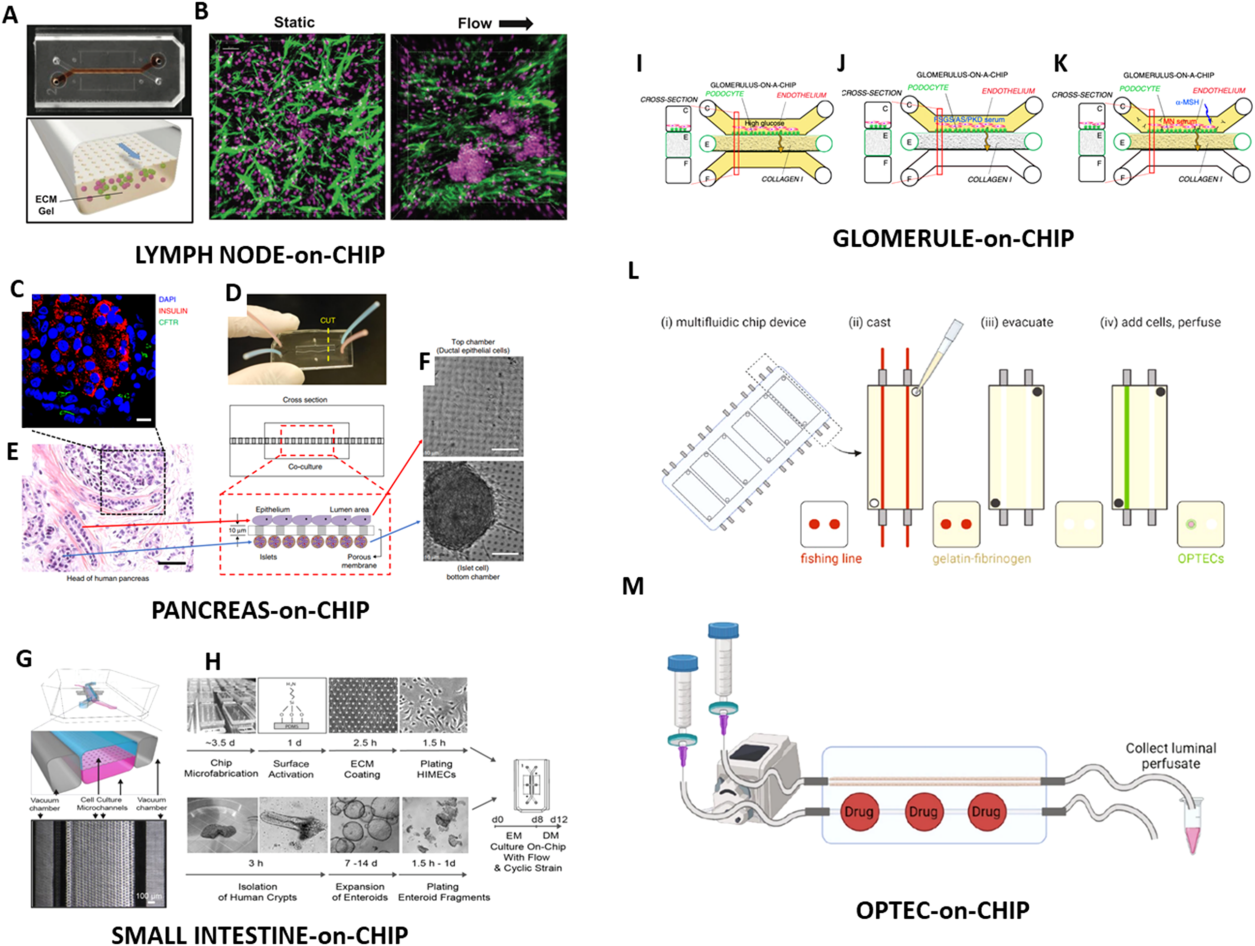
Autologous organ-on-chip models: *Lymph node-on chip*: **(A)** Digital picture (top) and cross-section scheme (bottom) of the chip; **(B)** evidence of many and larger multicellular aggregates of lymphocytes nuclei (magenta), and ECM fibers (green) aligned to flow direction, under flow condition compared to the static one; Reproduced with permission (162). *Design and validation of a patient-derived pancreas-on-a-chip model:***(C)** Immunofluorescence staining of a non-treated human pancreatic tissue section reveals the spatial proximity between insulin-producing islets and CFTR-expressing ductal structures; **(D)** Schematic representation of the pancreas-on-a-chip device, composed of two parallel microfluidic chambers separated by a porous membrane; **(E)** Histological analysis (H&E) confirms the structural relationship between islets and ducts in native tissue; **(F)** Within the chip, PDECs are cultured in the upper channel, while pancreatic islets are housed in the lower channel, enabling direct paracrine interaction*;* Reproduced under the terms of the Creative Commons Attribution 4.0 International License (163). *Primary human intestine-on-chip*: **(G)** schematic cross-sectional view (top) and a phase contrast micrograph of the chip viewed from above (bottom) showing the upper (epithelial; blue) and lower (microvascular; pink) cell culture microchannels separated by a porous, ECM-coated, PDMS membrane sandwiched in-between; **(H)** step-by-step fabrication for the establishment of microfluidic co-cultures of primary human intestinal epithelium and intestinal microvascular endothelium; Reproduced under the terms of the Creative Commons Attribution 4.0 International License (164). *Glomerulus-on-chip*: **(I)** Schematic representation of the albumin permselectivity assay, showing exposure of GOAC to increasing glucose concentrations (10, 15, 20 mM). After 72 hours, FITC-labeled albumin is introduced into channel C (yellow) and filtrate is collected from channel F. **(J)** Diagram of the GOAC exposed to 0.5% serum from individuals with FSGS, Alport syndrome (AS), or polycystic kidney disease (PKD), as well as healthy donors (CTRL1 and CTRL2). Albumin-FITC is applied after 24 hours and filtrate collected from channel F; **(K)** Schematic overview of the experimental design used to assess the effect of α-melanocyte-stimulating hormone (α-MSH) on albumin leakage in response to serum from a membranous nephropathy (MN) patient (MN3), with or without α-MSH Reproduced under the terms of the Creative Commons Attribution 4.0 International License (165). *OPTEC-on-chip*: **(L)** Schematic protocol layout to establish 3D OPTEC-on-chip models. **(M)** sketch of the experimental design used for drug administration and luminal sampling; Reproduced under the terms of the Creative Commons Attribution 4.0 International License (166).

An elegant example of a device developed to study the lymph node is the work carried out by Goyal and colleagues, using an OoC constituted by two channels separated by a porous membrane (**Figure 4A**) (162). The bottom channel was filled with primary human B and T cells, derived from peripheral blood, embedded in hydrogel (matrigel and collagen). The upper channel was used for medium perfusion. In this condition, the cells spontaneously self-assemble in structures resembling lymphoid follicles (LFs), with many larger multicellular aggregates compared to those obtained in static condition (**Figure 4B**). The LFs underwent antibody class switching, developed plasma cell clusters, and produced antigen-specific IgG upon antigen stimulation in the presence of dendritic cells. These chips served as a platform for evaluating the effectiveness of seasonal vaccines and adjuvants. Specifically, its usefulness on disease prevention was demonstrated by vaccinating the LF chips *in vitro* using a trivalent commercial influenza vaccine and a split H5N1 pandemic influenza antigen (A/turkey/Turkey/1/2005) along with a squalene oil-in-water adjuvant.

Another outstanding bone marrow model on chip is presented by Chou and colleagues (69). They used a two compartments device, realizing a non-autologous supportive “vascularized” channel, obtained by seeding human umbilical vein endothelial cells (HUVECs), and an autologous “hematopoietic” channel with a 3D co-culture of human CD34^+^ cells and bone marrow-derived mesenchymal stromal cells (BMSCs) in a fibrin gel.

In this study, immature CD16^lo^ neutrophils and CD71^+^CD235^−^ erythroid cells proliferated over four weeks and higher numbers of these neutrophil and erythroid precursors were found in the device than in suspension or static gel cultures. The microfluidic platform predicted clinically observed hematotoxicity at patient-relevant drug exposures. The perfusion of 5-fluorouracil (5-FU), a common chemotherapeutic agent, through the vascular channel of the device, consistently exhibited expected hematotoxicity at clinically relevant low micromolar concentrations across six donors of CD34^+^ progenitor cells, demonstrating the reliability and relevance for toxicity testing. In contrast, static suspension and gel culture failed to reproduce clinical toxicities at similar doses, only showing adverse effects at significantly higher concentrations (~40 μM), not aligned with patient-relevant conditions. Interestingly, the chip was also exploited with Shwachman–Diamond syndrome (SDS) patient cells, a genetic bone marrow failure syndrome characterized by biallelic mutations in the *SBDS* gene. Most patients exhibit hypocellular bone marrow and nearly all have neutropenia, also other blood cell lineages may also be affected, such as thrombocytopenia or anemia (167, 168). Thus, in the same work, CD34^+^ cells from SDS patients were cultured in the chip with normal BMSCs and ECs. After two weeks, the SDS chips showed significant hematopoietic defects and reduced cell development compared to the control (69). There was a notable decrease in both neutrophil and erythroid cells, reflecting the hypoplastic phenotype seen in SDS patients. CD34^+^ progenitor cell maintenance was impaired, aligning with observed intrinsic defects and reduced colony-forming ability in SDS patients.

### 6.3 Autologous pancreas-on-chip

Moving among genetic disorders, Mun et al. developed a patient-derived pancreas-on-chip to model cystic fibrosis-related disorders (163). They established an entirely autologous platform by isolating both pancreatic ductal epithelial cells (PDECs) and islets of Langerhans from the same individual who underwent total pancreatectomy with islet autotransplantation (TPIAT). Resected pancreatic tissue was enzymatically and mechanically dissociated to yield a remnant cell pellet containing islets (identified by dithizone staining) and ductal fragments, from which PDECs can be microdissected, embedded in matrigel, and expanded as 3D organoids. These organoids were coaxed into confluent monolayers expressing key epithelial markers, including keratin19 (KRT19), E-cadherin, epithelial sodium channel (ENaC), tight junction protein (like Zonula occludens-1 [ZO-1]) and cystic fibrosis transmembrane conductance regulator (CFTR), confirming their ductal identity. Functional assays demonstrated robust CFTR activity in both organoids (via fluid-secretion assays) and monolayers (short-circuit current measurements), and glucose-stimulated insulin release from islets was validated by ELISA. These two cell types were then cocultured in a microfluidic device (**Figures 4C-F**) composed of two PDMS chambers separated by a porous (10 µm) membrane; PDECs occupied the upper channel while islets resided below. Crucially, pharmacological inhibition of CFTR in the PDECs (using CFTRinh-172) led to a ~54% reduction in insulin secretion from the underlying islets, whereas direct application of inhibitor to islets alone had no effect, underscoring a ducto-insular cross-talk mediated by CFTR function. Together, these data provide the first direct evidence that CFTR activity in pancreatic ductal cells modulates endocrine function, and also establish a sensitive, reproducible, and wholly patient-derived *in vitro* system for investigating cystic fibrosis-related diabetes and testing personalized therapeutic interventions.

### 6.4 Autologous intestine-on-chip

Amidst the various organs modeled using OoC technology, the intestine has received significant attention. Traditionally, *in vitro* studies of intestinal biology have relied on immortalized cell lines, which, while informative, often fall short in recapitulating the intricate cellular interactions and physiological processes observed in the human intestine (169, 170). Exploiting the OoC technology, significant advancements can be made by integrating microfluidics, engineering and cell biology to create more physiologically relevant models of the intestine. These devices mimic key aspects of intestinal structures and functions, including epithelial barrier integrity, peristalsis-like forces and nutrients transport. Notably, the implementation of controlled apical or luminal flow dynamics has proven crucial for reproducing physiological conditions within intestinal chips. Shear stress generated by fluid flow not only promotes epithelial polarization and differentiation but also enhances tight junction expression and maintains mucus layer stability and secretion (144, 171). As for other OoC model, the choice of the cell source is critical. While immortalized cell lines like Caco-2 and T84 cells have limitations in representing individual-specific responses and complex behaviors due to their transformed nature, primary intestinal cells derived directly from human donors or patients offer several advantages, as previously mentioned. Recently, different models have been presented, focusing on the recapitulation of the human-microbial interaction, as well as on the exploitation of human and patient-derived intestine organoids creating aOoC. In 2018 Ingber’s group presented a small intestine-on-chip using biopsy-derived organoids (164). Epithelial cells were isolated from healthy region of human biopsies, expanded as 3D organoids, dissociated and cultured on a device characterized by an architecture consisting of a classical design featured by two microchannels separated by a porous membrane (**Figures 4G, H**). In such a condition, polarized epithelial cells with villi-like projections reconstituted a microenvironment strictly similar to *in vivo* duodenum, as revealed by transcriptomic analysis. Assessed morphology and gene expression, the functionality of the system based on biopsy-derived cells was tested evaluating the ability to replicate normal intestine function. Chips based on cells from different donors exhibited proper enzymatic activity and expression of mucin-2 (MUC2), which is responsible for production of mucus the line the gut.

More recently, Shin et al. reported the development of patient-specific, organoid-based physiodynamic mucosal interface-on-a-chip (172). In this study, the intestinal microenvironment was recapitulated by applying defined mechanical deformations and fluid hydrodynamics, exploiting a coculture with a host microbiome while allowing the real-time monitoring of cell morphology and cell-cell interactions, in a manner not possible with a standard organoid culture. Interestingly, Good and Warner groups collaborated moving the focus on the vasculature of intestine tissue using patient-derived small intestinal subepithelial myofibroblasts (ISEMFs) and primary ECs (173). They designed a microfluidic device able to support the growth of patient-derived ISEMFs and ECs, demonstrating the response of the system to different physiological parameters, such as oxygen tension, cell density, growth factors. Then, the response of the system to growth factors like vascular endothelial growth factor (VEGF), insulin-like growth factor (IGF), epidermal growth factor (EGF) and fibroblast growth factor (FGF) was evaluated, to both assess the pro-angiogenic effect and the absence of undesired off-target effect. The optical transparency of the device allowed an accurate measurement of the tight junctions’ number, the endothelial cell expansion and the total vessel length under growth factor perfusion. Moreover, the effect of a drug was tested: Erlotinib was selected as anti-angiogenic molecule, clinically used as a synergistic anti-neoplastic agent. Adding Erlotinib to the established vasculature, the vessel remodeling was prevented. In parallel, the authors also tested the combined effect of ISEMFs and ECs on patient-derived human intestinal epithelial cells (HIECs). In fact, HIEC proliferation and TEER significantly increases when co-cultured simultaneously with both cell types, advocating for the importance of developing complex OoC systems that can generate more biologically relevant outputs.

In another study presented by Ingber’s group in 2022 (174), environmental enteric dysfunction (EED), a chronic inflammatory condition of the intestine, was recapitulated on a microfluidic device lined with organoid-derived intestinal epithelial cells from EED patients. Thanks to the access to two parallels flow channels, the device allowed the quantification of the intestinal barrier function, as well as the measurement of the transepithelial absorption and transport. Moreover, the effect of nutritional deficiencies on this disease model, from both a phenotypic and functional point of view, were able to be explored. Thus, the intestine-on-chip was able to recapitulate characteristic EED signatures, like upregulation of antimicrobial genes and downregulation of metallothioneins and genes involved in digestion and metabolism, providing a reliable tool for the disease study and drug testing, as well for developing a personalized therapy. In summary, OoC technology offers a promising approach to bridge the gap between traditional cell culture models and *in vivo* studies of human intestinal physiology.

### 6.5 Autologous kidney-on-chip

OoC technology has also been applied to model the kidney, a key organ of the excretory system that regulates fluid balance, electrolyte homeostasis, and the elimination of metabolic waste. Because kidney dysfunction has severe clinical implications, there is a strong need for physiologically relevant platforms to study renal physiology and disease mechanisms (175).

Conventional 2D cultures and animal models struggle to reproduce the kidney’s intricate architecture and dynamic transport processes. Kidney-on-chip systems were developed to overcome these limitations. As observed in other organ models, most reported kidney-on-chip studies still rely on immortalized cell lines (176–180), although recent work has increasingly explored patient-derived primary cells, spheroids, and organoids to establish autologous renal chips (166, 181–185). In 2013, *in vivo*-like pathophysiology studies were carried out cultivating primary commercial human proximal tubular epithelial cells on an OoC exploiting it for a toxicity study (165). Petrosyan et al. developed a glomerulus-on-a-chip (GOAC) by reconstructing the glomerular filtration barrier on a microfluidic chip using human podocytes and glomerular endothelial cells (hGEC). This device was effective in maintaining cell morphology over long-term culture, demonstrating characteristic glomerular structure and function. Moreover, the authors used three different types of podocytes: primary podocytes from healthy donors (hpPOD), an immortalized podocyte cell line (hiPOD) and podocytes derived from amniotic fluid (hAKPC-P). All three were able to be co-cultured with primary hGEC from the same donor as hpPOD inside the GOAC, although hiPOD evidenced a less organized nephrin distribution, lower secretion of matrix proteins, such as collagen IV and laminin, and lower efficiency of permselectivity, which exalts the relevance of shifting away from using cell lines in OoC models. The model’s validation involved the use of serum from individuals with different chronic kidney diseases (CKD), such as membranous nephropathy, and assessing drug responses (184). The chip was tested in modelling diabetic nephropathy and a glucose-induced damage was revealed compared to a control group (**Figure 4I**). Importantly, the specificity of response to sera from patients with different CKD (**Figures 4J, K**), like focal segmental glomerulosclerosis (FGS), membranous nephropathy (MN), Alport syndrome (AS) and polycystic kidney disease (PKD) was tested, and the chip reacted similarly to human glomeruli in response to nephrotoxic serum and nephroprotective treatment, mirroring *in vivo* conditions.

In the same year, Hans Clever’s group presented a tubuloids-on chip model (165, 166, 182–186). Adult stem cell-derived organoids have emerged as powerful tools for studying organ-specific biology and disease modeling and they achieved a long-term culture (over 6 months) of primary kidney tubular epithelial organoids, named “tubuloids”. These human tubuloids encompassed both proximal and distal nephron segments, as confirmed by gene expression profiling, immunofluorescence studies, and functional tubular analyses. A diverse array of kidney conditions including infectious, malignant, and hereditary diseases, was screened in a personalized manner, and once cultured in an OoC platform, tubuloids exhibited a tubular structure and demonstrated active epithelial transport function, showcasing their potential for advanced disease modeling and drug screening applications. Later, in a similar manner, Aceves et al. presented a work based on organoid‐derived proximal tubule epithelial cells (OPTEC)-on-chip, providing a more sensitive predictor of nephrotoxicity compared to traditional models based on immortalized PTECs (166). The innovative approach involved the isolation of PTECs from kidney organoids, seeding them into a 3D microenvironment that emulates native tubular structures (**Figure 4L**). The cells exhibited the desired cuboidal morphology and efficiently formed a confluent epithelial monolayer when organized into 3D tubules. Upon perfusion, OPTEC tubules displayed proper polarization and expressed a broad array of proximal-tubule-specific functional markers, exhibiting higher expression levels of basolateral drug transporters, namely organic cationic/carnitine transporter 2 (OCT2), organic anion transporter 1 (OAT1) and OAT3, compared to immortalized PTECs. This 3D OPTEC-on-chip model was used to investigate the polarized drug uptake via organic cation and anion transporters. Cisplatin, used in chemotherapy, and aristolochic acid, widely diffused in the Chinese herbal medicine, known to induce nephrotoxic effect, were perfused and the OPTEC-on-chip revealed to be a more reliable *in vitro* system compared to PTEC (**Figure 4M**). Thus, developing an autologous kidney-on-chip instead of a model based on immortalized cell lines is crucial for accurately replicating patient-specific renal physiology and disease mechanisms. Immortalized cell lines, while convenient and reproducible, often lack the functional complexity, genetic diversity, and physiological responses of primary kidney cells, limiting their ability to model individual variations in renal function and pathology.

## 7 Autologous multi-tissue OoC models: toward personalized medicine through applications and challenges

While many diseases are associated with dysfunction in a single tissue, their progression and therapeutic outcomes are fundamentally shaped by systemic and inter-organ interactions. Pathophysiological processes, such as inflammation, fibrosis, metabolic imbalance, and immune modulation, frequently arise from dynamic crosstalk among multiple cell types originating from different tissues. For example, metabolic disorders like type 2 diabetes involve a complex interplay between pancreatic, hepatic, adipose, and immune cells (187), while cancer progression and drug resistance are increasingly linked to interactions between tumor cells, stromal fibroblasts, immune infiltrates, and endothelial components (188, 189).

Traditional single tissue OoC systems, while valuable, fail to capture this intricate biological interdependence. Autologous multi-tissue OoC platforms, by integrating multiple primary or stem cell–derived cell types from the same individual within interconnected microphysiological compartments are the new frontier, since they offer a powerful approach to faithfully replicate a specific pathology including in the very same model both inter-organ interactions and patient-specific physiology.

### 7.1 Leukemia-on-chip

**Figure 5:**
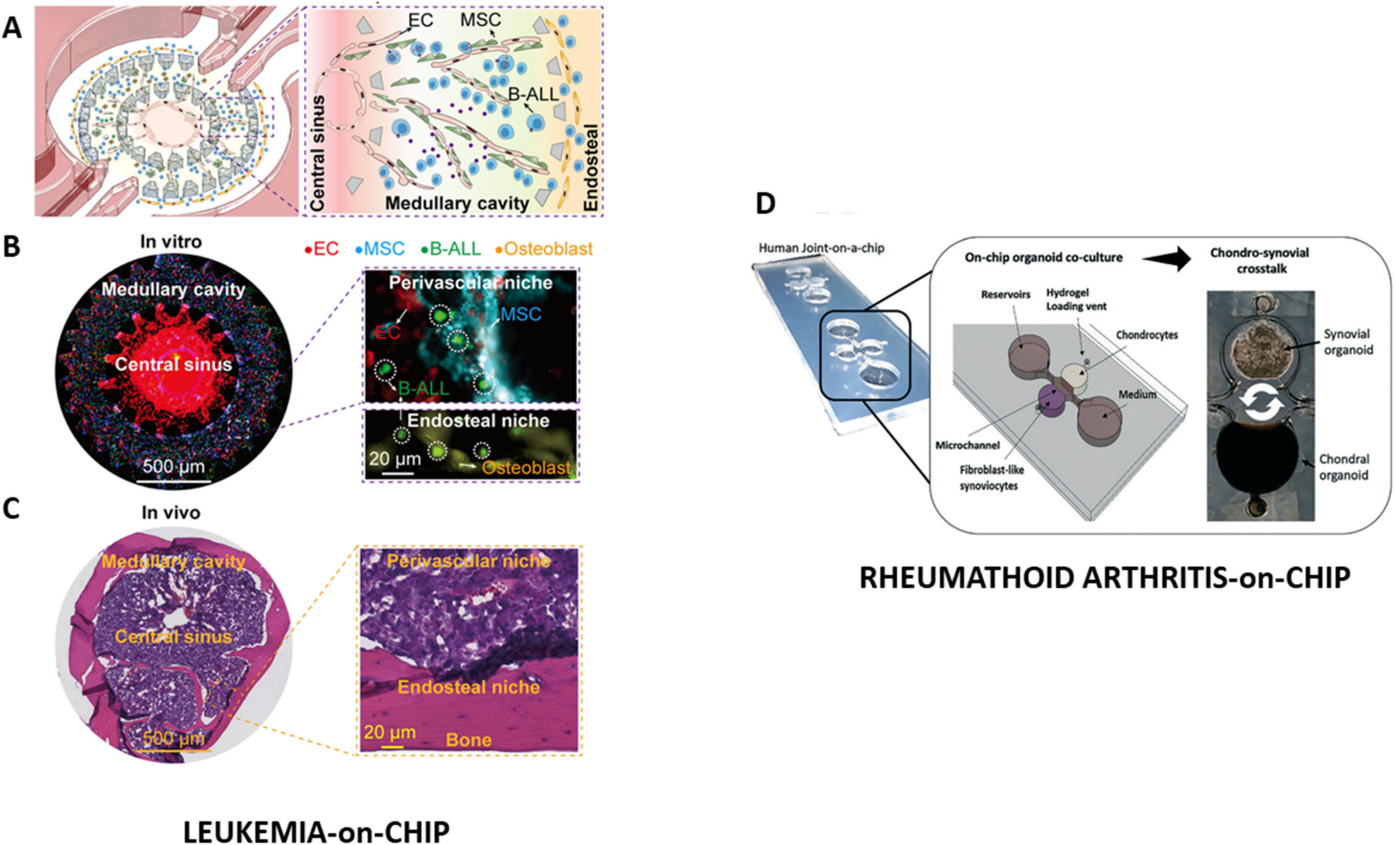
Autologous multi-tissue organ-on-chip models*: Leukemia-on-chip*: **(A)** Graphic representation of the three functional regions; **(B)** Complete imaging of the on chip leukemic bone marrow niche reveals B-ALL cells gathered in perivascular areas. The bottom inset highlights their presence in the endosteal niche; **(C)** Hematoxylin and eosin (H&E) staining image of the *in vivo* leukemic bone marrow niche with B-ALL cells localized within both perivascular and endosteal niches, as highlighted in the enlarged view. Reproduced under the terms of the Creative Commons Attribution 4.0 International License (190). *RA Joint-on-chip*: **(D)** Schematic representation of the organoid-based joint-on-a-chip system featuring chondral and synovial compartments designed to model reciprocal inflammatory interactions; Reproduced under the terms of the Creative Commons Attribution 3.0 International License (191).

In 2020, Ma et al. presented a leukemia-on-chip for studying B-cell acute lymphoblastic leukemia (B-ALL), the most common cancer among children, characterized by the overproduction of immature and dysfunctional B-cell blasts within bone marrow (190). They proposed a microfluidic platform constituted of three regions in which patient-derived B-ALL cells from different subtypes, together with primary commercial HUVECs, mesenchymal stem cells (MSC) and osteoblasts, were seeded for mimicking the human cytological and histological complexity of bone marrow (**Figures 5A-C**). In this way the interaction between bone marrow and leukemia microenvironment was recapitulated. Thus, thanks to the control on biological parameters (cells composition and organization), real-time monitoring and gradients control allowed by the microfluidic device, the chip was used to investigate the mechanisms underlying the progression and chemoresistance of such non-solid tumor. The dynamic interactions between B-ALL blasts and niche cells (vascular ECs, perivascular mesenchymal stem cells, and osteoblasts) were studied, identifying specific roles of these cells in regulating cytokines, adhesive signaling, and downstream B-ALL survival pathways, as well as markers of proliferation and quiescence, highlighting a subtype-related heterogeneity and responses to treatment. Moreover, the leukemia-on chip allowed the study of the spatial heterogeneity in the leukemic niche as well as to investigate the mechanisms underlying the leukemia survival across different patient-derived samples. This platform can be suitable for other hematological malignancies representing a precious tool for pathophysiology study and in the precision medicine context.

### 7.2 Rheumatoid arthritis-on-chip

Further, among the pathologies in which multi-tissue crosstalk plays a pivotal role, RA stands out as a paradigmatic example. During disease progression, circulating immune cells such as monocytes and lymphocytes infiltrate the synovial membrane, triggering a cascade of pro-inflammatory signals that manifest clinically as joint swelling. These infiltrating immune cells, in concert with fibroblast-like synoviocytes (FLS) and resident macrophages, promote chronic inflammation and stimulate the release of ECM-degrading enzymes and key factors such as receptor activator of NF-κB ligand (RANKL), which enhance osteoclast differentiation and activity (192). As a result, cartilage and bone are progressively degraded, leading to joint instability, impaired movement, and long-term permanent disability. Despite decades of research, RA’s etiology remains only partially understood, and current therapeutic strategies, including biologic agents, often show variable efficacy and high failure rates across patients (67, 193). Thus, there is a growing need to implement innovative strategies for assessing drug efficacy more rapidly and with greater personalization, highlighting the urgent need for advanced *in vitro* models, including the immune-competent, multi-tissue RA-on-chip platforms, capable of replicating the complex interplay between immune cells and joint-resident stromal components (49, 94, 194, 195).

As a representative example of this advanced *in vitro* approach, in 2021 Peter Ertl’s group established a synovium-on-chip platform that leverages patient-derived FLS. These cells were embedded in matrigel to form 3D organoids, enabling the study of inflammatory responses and tissue remodeling processes within a controlled microfluidic environment. Through optical light-scatter biosensing, architectural changes derived from TNF-α-induced inflammation were reliably detected in a non-invasive manner, while maintaining a stable culture for over a week (39). The same authors also established a dual organoid model where RA-patient-derived synovial organoids were cocultured with chondral organoids, originated from commercial primary chondrocytes (46). In this joint-on-a-chip platform integrates patient-derived FLS embedded in matrigel and primary human articular chondrocytes embedded in fibrin hydrogel, housed in spatially separated but communication-enabled microchambers (**Figure 5D**). Key design improvements ensured high reproducibility of organoid size, morphology, and spatial positioning, establishing a reliable platform for controlled tissue-tissue interactions. Notably, the study demonstrates that co-culture with synovial organoids stabilizes chondrocyte morphology and supports more cartilage-like phenotypes. To study how cell differences relate to organoid condensation, researchers stained mono- and co-cultured organoids with a red-fluorescent dye (CMTPX) at day 0 and observed them for 3 weeks. Over time, chondrocytes with a round shape decreased in monocultures but were better maintained in co-culture with synovial organoids, indicating enhanced morphological stability in co-culture conditions.

This model is missing some key elements to mimic RA pathobiology, like vascular structures and PBMCs. *In vivo*, blood vessels do more than just deliver oxygen and nutrients; they also create pathways for PBMCs to extravasate from bloodstream into tissues. Understanding this process of extravasation is vital for grasping how immune cells travel, how inflammation occurs, and how tissues respond. Additionally, having functional blood vessels is essential for drug testing how well they can be delivered and how effective they are in conditions that closely mimic the real body.

### 7.3 Osteoarthritis-on-chip

Although RA and osteoarthritis (OA) differ in etiology and pathogenesis, both are joint-related diseases involving complex interactions between synovial and cartilage tissues. In this framework, Petta et al. developed a personalized joint-on-a-chip platform that integrates human chondrocytes and FLS isolated from OA patients into a compartmentalized microfluidic device (38). The model recreates essential joint compartments, cartilage, synovium, and synovial fluid, allowing for a 3D coculture system within biomimetic hydrogels, particularly hyaluronic acid-poly(ethylene glycol) diacrylate (HA-PEGDA) for chondrocytes and fibrin for FLS. Upon addition of patient-matched OA synovial fluid, the model reproduces key disease features, including cellular senescence, upregulation of inflammatory cytokines (e.g., IL-6, IL-8, TNF-α), and expression of matrix-degrading enzymes (matrix metalloproteinase [MMP] 1 and MMP13), effectively mimicking the catabolic OA microenvironment. Notably, in a proof-of-concept application, the authors used this chip to evaluate the therapeutic potential of allogeneic MSCs (from bone marrow or adipose tissue) injected into the central fluidic channel. Results revealed patient-specific responses in cytokine modulation and MMP expression, underscoring the system’s capacity to function as a personalized screening tool for orthobiologic therapies. This study exemplifies the potential of autologous, multi-tissue OoC models to stratify patients and tailor treatments for complex joint diseases such as OA.

### 7.4 Autologous vascular compartment: a challenge toward immunocompetent aOoC systems

Significant progress has been made in developing sophisticated OoC platforms for joint pathology, yet a fundamental physiological component remains insufficiently represented: the vascular network. Given its essential functions in nutrient exchange, immune surveillance, and inflammatory signaling, the limited incorporation of autologous, perfusable vasculature constitutes a critical barrier to recapitulating joint pathophysiology *in vitro*. Importantly, this limitation is not restricted to musculoskeletal models, but reflects a broader challenge within aOoC research, where reconstructing patient specific vascularized niches remains a major biological and engineering frontier.

To date, most models rely on commercial ECs such as HUVECs due to their availability and ease of use (145, 196). However, this choice comes with important limitations. HUVECs are derived from large vessels of the umbilical cord and represent only one subtype of ECs, while different tissues possess specialized EC populations with unique immunomodulatory roles. As reviewed by Amersfoort et al., the tissue-specific nature of ECs is critical for accurately recapitulating organ physiology and immune interactions (197, 198). In the context of aOoC models incorporating patient-derived immune cells, the use of mismatched commercial ECs, sourced from unrelated donors, can provoke non-specific immune activation. HUVECs, in particular, act as semi-professional antigen-presenting cells, expressing major histocompatibility complex (MHC) class I constitutively and MHC class II in an inducible manner, thus potentially enhancing T-cell activation and distorting disease modelling (199, 200).

To avoid such immune mismatches and improve the physiological relevance of aOoC platforms, it is essential to develop autologous vascular components. While generating patient-specific ECs from iPSCs represents one viable strategy (201), this approach is time-consuming and may introduce genetic and epigenetic changes. Yet, promising efforts have begun to explore the use of freshly isolated, healthy ECs in chip systems. For instance, Phan et al. developed a microfluidic platform containing three tissue chambers lined with endothelial colony-forming cell-derived ECs (ECFC-ECs) from cord blood, flanked by perfusion channels (202). Their system yielded reproducible, functional vascularized microtissues, which supported microtumour formation and facilitated accurate anti-cancer drug screening with Food and Drug Administration (FDA)-approved compounds.

Therefore, integrating patient-specific vasculature is crucial for advancing immune-competent aOoC systems and ensuring reliable disease modelling, particularly in immune-mediated and inflammatory disorders.

### 7.5 Co-culture-on-chip of multiple autologous cells: a challenge toward multi-tissue aOoC

Another major challenge in the development of multi-tissue aOoC platforms lies in the integration and functional synchronization of different autologous cell types within a shared microphysiological environment. In many disease models, such as those representing joints, metabolic organs, or immune niches, replicating *in vivo*-like crosstalk requires the co-culture of distinct patient-derived cells, including epithelial, stromal, immune, and vascular components. However, these cell types often exhibit divergent requirements for media composition, mechanical stimuli, oxygen tension, and ECM cues, making it difficult to maintain their viability and phenotypic stability within a single device (203). Moreover, the timing of differentiation, seeding, and maturation must often be finely tuned to ensure appropriate cellular organization and function, especially in systems that mimic tissue development or disease progression. This complexity is compounded in autologous models, where cell yields can vary widely depending on the patient’s health status and tissue availability, limiting scalability and reproducibility. Overcoming these issues will require the development of adaptive microfluidic architectures, compartmentalized culture strategies, and modular biomaterial systems that can support parallel yet intercommunicating tissue environments, tailored to each cell type’s specific requirements while preserving systemic integration. **Table 2** summarizes the critical parameters, categorized for systematic evaluation.

**Table 2:** Key parameters for the design, operation, and validation of multi-organ-on-a-chip systems.

Category	Key parameter	Description and rationale	References
Platform design	Interconnection strategy	Defines how tissues communicate (e.g., shared medium, partitioned flow with permeable barriers like membranes or pillars). Critical for modeling systemic transport and paracrine signaling.	(191, 204–206)
	Physiological scaling	The ratio of functional cell numbers or tissue sizes between compartments. Aims to mimic *in vivo* organ mass ratios or functional capacities (e.g., metabolic clearance vs. absorptive surface).	
	Fluidic regime	The flow characteristics (e.g., continuous, pulsatile, static). Controls shear stress, nutrient/waste exchange, and molecular distribution.	
Biological components	Cell source	Origin of cells for each tissue unit (e.g., primary cells, iPSC-derived, cell lines). Determines genetic background, functionality, and relevance to human physiology.	(38, 197, 199, 201)
	Tissue maturity	The degree of differentiation and functionality (e.g., albumin production in liver models, TEER in barrier tissues) prior to and during co-culture.	
Functional output and validation	Tissue-specific function	Quantitative metrics for each organ’s health and specialized activity (e.g., Albumin/Urea production (liver), TEER/Permeability (barriers), Beating Rate (heart), Spiking Activity (neurons).	(48, 207–209)
	Pharmacokinetic profile	Measurement of systemic ADME processes: Absorption, Distribution, Metabolism, and Excretion. Key metrics include AUC, Clearance, Metabolite Formation, and Terminal Half-life (t½).	
	Systemic toxicity	Assessment of adverse effects on non-target tissues, demonstrating the platform’s ability to predict organ-specific toxicity (e.g., viability loss, LDH release in secondary organs).	
	Inter-tissue crosstalk	Direct evidence of communication, measured via cytokine/hormone signaling or a functional response in a distal tissue to a stimulus applied to another.	

## 8 Assessing predictive capacity: validation against animal models and clinical data

The translational promise of OoC technology is fundamentally contingent upon its demonstrated predictive validity for human physiology and pathology. For OoCs to transition from sophisticated research tools to reliable preclinical platforms, their output must be quantitatively benchmarked against gold-standard *in vivo* data (210). This requires moving beyond proof-of-concept demonstrations of viability and function to systematic comparisons of pharmacokinetic profiles, toxicological endpoints, and disease treatment responses against existing animal model data and, crucially, human clinical outcomes. Key validation metrics include pharmacokinetics/pharmacodynamics concordance (e.g., clearance rates, area under the curve [AUC], metabolite formation), toxicological sensitivity and specificity, and the recapitulation of clinical-grade disease phenotypes and drug responses.

A growing, though still nascent, body of evidence provides compelling cases for OoC predictivity. For instance, a landmark study on a human gut-liver-kidney on chip demonstrated the accurate replication of human-specific pharmacokinetics of cisplatin, predicting its dose-dependent nephrotoxicity which is a human-relevant outcome that can be poorly predicted by rodent models (211). Further, Jang et al. demonstrated the strong translational potential of microengineered liver-on-chip platforms capable of reproducing species-specific hepatotoxic responses across rat, dog, and human models (212). By integrating primary hepatocytes with non-parenchymal liver cells under continuous perfusion, the human liver-on-chip maintained long-term metabolic competence and reproduced clinically relevant mechanisms of drug-induced liver injury (DILI) at exposure levels comparable to patient plasma concentrations. Importantly, it enabled discrimination between human-specific and animal-specific toxicities, such as those induced by bosentan (an endothelin receptor antagonist with known dose-dependent human hepatotoxicity) and fialuridine (an antiviral nucleoside analog that caused fatal human liver toxicity undetected in animals), thereby improving the extrapolation of preclinical findings to human outcomes. Beyond traditional viability assays, the system captured mechanistic endpoints including mitochondrial dysfunction, oxidative stress, and fibrosis-related pathways, highlighting its suitability for mechanistic studies, biomarker discovery, and safety risk assessment.

Not only toxicology, OoCs are being validated against patient-specific clinical data. In a recent study Maulana et al. introduced a patient-derived breast cancer-on-chip model designed to emulate key features of the human tumor microenvironment while enabling personalized assessment of immunotherapy efficacy and safety (213). The platform combines primary tumor cells from breast cancer patients with a microvascular endothelial layer and circulating immune cells within a perfused microfluidic system, recreating dynamic interactions between immune and tumor compartments. The model allows real-time monitoring of immune cell trafficking, endothelial transmigration, cytokine secretion, and tumor cell killing under physiologically relevant flow conditions. Importantly, it revealed inter-patient variability in response to chimeric antigen receptor (CAR)-T-cell therapy, capturing both effective tumor eradication and excessive cytokine release patterns that mirror clinical immune-related toxicities.

The effectiveness of immunotherapies relies on the ability of immune cells to reach, infiltrate, and kill cancer cells, features that conventional *in vitro* cultures and animal models fail to accurately reproduce. To address this gap, Margazalli et al. developed a humanized OoC platform to model natural killer (NK) cell behavior under physiological flow (214). In this system, circulating NK cells spontaneously extravasated toward a physically separated 3D neuroblastoma niche, infiltrate the tumor matrix, and induce tumor apoptosis, while showing a reduced proportion of CD16^+^ cells within the migrated/infiltrated population, an observation relevant to clinical prognosis and therapy response.

By aligning *in vitro* responses with known patient-specific outcomes, these studies underscore the translational potential of OoC platforms for studying immune recruitment, supporting more reliable testing of immunotherapies and potential personalization. These are some examples of how OoC technology can represent a next-generation preclinical testing tool, moving beyond conventional toxicology toward precision immuno-oncology, where patient-derived chips could guide therapeutic decisions and dose optimization while reducing reliance on animal models.

## 9 The emerging role of millifluidic platforms in translational applications

While OoC platforms excel at replicating microscale tissue architectures and physiological shear stresses, their characteristic small volumes (nanoliters to microliters) can pose significant challenges for translational research. These include constrained tissue sizes, difficulty in cell retrieval for downstream analysis, and, most critically, a limited volume of effluent for comprehensive molecular profiling (e.g., proteomics, metabolomics), which is essential for biomarker discovery and mechanistic studies (54). To bridge this gap between conventional microfluidics and macroscale bioreactors, millifluidic systems have emerged as a powerful intermediate category. Characterized by channel dimensions of hundreds of micrometers to millimeters and working volumes from hundreds of microliters to milliliters, these platforms offer distinct advantages for specific translational contexts (215–218).

The primary operational benefit of millifluidic systems is their substantially larger fluid and chamber volumes. This scale enhances sample availability, allowing for frequent and substantial collection of conditioned media for multi omic analyses without perturbing the system’s homeostasis, thereby furnishing a richer dataset for robust biomarker identification and pharmacokinetics/pharmacodynamics modeling. The increased physical dimensions also simplify tissue handling and cell harvesting, enabling straightforward access to constructs for endpoint analyses, for example histology and RNA sequencing, using standard laboratory protocols. This operational simplicity, in turn, fosters superior clinical integration, as millifluidic devices interface more seamlessly with established workflows such as automated liquid handlers, lowering the barrier to adoption in pharmaceutical screening.

It is important to note that millifluidic platforms complement, rather than replace, their microfluidic counterparts. They are particularly well suited for applications requiring robust, high fidelity tissue models that generate sufficient material for deep molecular phenotyping, rather than replicating precise capillary scale flow dynamics. For instance, they have been successfully used to culture large, patient derived organoids and tissue slices (217). However, one limitation of patient-specific millifluidic systems is their reliance on primary tissue procurement, since the number of cells obtainable from minimally invasive biopsies is often limited. This constraint may reduce experimental throughput and necessitate optimization strategies to maximize the use of scarce patient-derived material.

In conclusion, incorporating millifluidic architectures into the organ on chip toolkit acknowledges that a one size fits all approach is insufficient. By offering a pragmatic balance between physiological relevance and analytical practicality, millifluidic systems can present a compelling pathway to overcome key bottlenecks in sample yield and workflow integration, thereby accelerating the deployment of these advanced models in drug development and personalized medicine.

## 10 Concluding remarks and future perspectives

OoC technology has emerged as a powerful tool for modeling human physiology and disease, offering a more physiologically relevant alternative to traditional *in vitro* and animal models. Among these, aOoC systems represent a significant advancement, integrating patient-derived target and immune cells to create personalized microenvironments that profoundly enhance disease modeling, drug screening, and regenerative medicine applications.

The potential of this technology extends to providing a promising alternative to conventional clinical trials. By replicating human organ functions using patient-derived cells, these autologous systems can model individual responses to therapies, potentially reducing reliance on traditional trials. Hypothetically, in oncology, tumor-on-chip platforms derived from a patient’s cancer cells could be used to identify the most effective chemotherapeutic or immunotherapy combinations *in vitro*, minimizing systemic toxicity. For complex multifactorial diseases such as autoimmune disorders, aOoC systems could facilitate the discovery of personalized biomarkers and therapeutic targets by recreating each patient’s unique genetic, epigenetic, and microenvironmental landscape. This capability could further facilitate the integration of on-chip data with high throughput omics analyses and artificial intelligence, enabling the convergence of biological twins with dynamic digital twins that predict disease progression and treatment responses in real time. Moreover, multi-organ autologous chip platforms could replicate critical inter-organ communication, allowing for a more complete understanding of systemic drug responses and adverse effects. Overall, the aOoC approach holds great promise for ushering in a new era of precision medicine, enabling tailored therapeutic strategies and reducing the risks associated with generalized pre-clinical models.

However, several challenges must be overcome for their widespread adoption. Technical hurdles include the fine-tuning and scalability of fabrication techniques, the optimization of cell sourcing and differentiation protocols, and the integration of real-time biosensors. Ensuring the long-term viability and functionality of patient-derived cells within microfluidic environments also remains a key obstacle. Furthermore, the miniaturized scale of OoCs, while advantageous for mimicking microenvironments, limits the variety of obtainable readouts. Common techniques, such as transcriptomics and proteomics, require significantly more starting material than what a single chip can typically provide, necessitating multiple replicates for a single analysis. Similarly, the quantity of secreted factors, like cytokines and EVs collected from the flow-through, can be scarce for robust downstream analysis.

To address these technical limitations and enhance reliability, the field must implement concrete strategies, including standardized quality control metrics, defined minimum numbers of biological and technical replicates, and data pooling approaches across identical experimental setups. The development and adoption of harmonized standard operating procedures for device fabrication, cell culture, and assay performance are critical for improving comparability across laboratories.

A coordinated, multi-level effort is underway to establish the necessary technical and regulatory frameworks. Recognizing this need, several initiatives are actively establishing validation frameworks. Since 2013, the European Commission has launched the Putting Science into Standards (PSIS) initiative, which led in 2021 to a thematic workshop on OoC and later to the creation of a dedicated Focus Group of experts (FG OoC) (219, 220). In 2024, this group drew up a comprehensive standardization roadmap. Recently, worldwide efforts culminated in the creation of an ISO technical sub-committee (ISO/TC 276/SC 2) specifically tasked with identifying standardization needs and gaps in the field of microphysiological systems and OoC. These institutional efforts are complemented by community-driven initiatives such as the Organ-on-Chip Development Project (ORCHID) and its successor, the European Organ-on-Chip Society (EUROoCS), which have been instrumental in advancing the integration and standardization of OoC technologies within Europe’s biomedical infrastructure.

In parallel, regulatory bodies are developing pathways for the qualification of OoC technologies. The U.S. FDA, through its Modernization Act 2.0 and 3.0, promotes the use of advanced non-animal models (221). Its Innovative Science and Technology Approaches for New Drugs (ISTAND) pilot program is explicitly considering complex *in vitro* models like OoCs, with a liver-on-chip for DILI being the first OoC accepted into the program for evaluation (222). Similarly, the European Medicines Agency (EMA) has published reflections on the qualification of novel methodologies, providing a conceptual framework for their evaluation (223). International consortia like the Organization for Economic Co-operation and Development (OECD) are also exploring the standardization of these systems for specific toxicological endpoints (224). This multi-staged, collaborative approach between developers, regulators, and academic partners is critical to building the evidentiary basis required for OoCs to be accepted as trustworthy preclinical or diagnostic tools. As this occurs, we see the progressive integration of specific platforms: cardiovascular OoCs are serving as complementary tools, while blood-brain barrier-on-chip models are rapidly progressing to decrease dependence on animal models in neurological research.

In conclusion, collaborative efforts among researchers, clinicians, industry, and regulatory bodies are essential to integrate these platforms into the drug development process. Following the necessary technical standardization and regulatory qualification, the path for OoCs to transition from sophisticated research tools to mainstream pre-clinical assets becomes clearer. As these technologies continue to evolve and overcome these critical hurdles, they are poised to fundamentally transform drug discovery, toxicology testing, and precision medicine. Crucially, by generating patient-specific data prior to clinical enrollment, these systems can inform trial design, identify likely responders, and support patient stratification, thereby complementing and reducing the risks associated with clinical trials. The ability to recapitulate individual human pathophysiology in a controlled system will not only advance personalized therapeutic strategies but also enable trial-on-chip approaches that anticipate clinical responses and reduce exposure to ineffective treatments. Ultimately, this progress will foster more predictive, efficient, and patient-centered clinical development pathways and contribute to a more human relevant healthcare paradigm.
